# *Toxoplasma gondii* in African Wildlife: A Systematic Review

**DOI:** 10.3390/pathogens11080868

**Published:** 2022-08-01

**Authors:** Refilwe Philadelphia Bokaba, Veronique Dermauw, Darshana Morar-Leather, Pierre Dorny, Luis Neves

**Affiliations:** 1 Department of Veterinary Tropical Diseases, University of Pretoria, Private Bag X04, Onderstepoort, Pretoria 0110, South Africa; darshana.morar-leather@up.ac.za (D.M.-L.); luis.neves@up.ac.za (L.N.); 2 Department of Biomedical Sciences, Institute of Tropical Medicine, 2000 Antwerp, Belgium; vdermauw@itg.be (V.D.); pdorny@itg.be (P.D.); 3 Centro de Biotecnologia, Universidade Eduardo Mondlane, Maputo 3453, Mozambique

**Keywords:** *Toxoplasma gondii*, wildlife, prevalence, Africa

## Abstract

*Toxoplasma gondii* (*T. gondii*) is a protozoan parasite, which infects a wide variety of mammals and bird species globally. In large parts of the world, this parasite is relatively well documented in wildlife species, however, this topic is poorly documented in Africa. The current review systematically explores the presence and distribution of *T. gondii* in African wildlife species through a key word search in PubMed, Web of Science and CAB Direct. A total of 66 records were identified and included in the qualitative analysis, of which 19 records were retained for the quantitative synthesis. The presence of *T. gondii* was reported in a wide range of wildlife species, found in twelve countries, spread over the African continent. The retained records report a prevalence range of 6–100% in herbivores, 8–100% in omnivores and 14–100% in carnivores. In wild felines (cheetahs, leopards, and lions) a prevalence range of 33–100% was found. Reports from South Africa, and on the presence of *T. gondii* in lion were most common. Overall, the results indicate the scarcity of information on *T. gondii* in Africa and its circulation in wildlife. The lack of knowledge on the parasite in Africa, especially in areas at the human-livestock-wildlife interface, prevents us from understanding how prevalent it is on the continent, what strains are circulating in wildlife and what the most common routes of transmission are in the different habitats in Africa.

## 1. Introduction

*Toxoplasma gondii* (*T. gondii)* is an apicomplexan protozoan parasite that causes a zoonotic infection known as toxoplasmosis. This parasite is one of the most resilient and persistent parasites in existence, able to infect a large diversity of homeotherms worldwide [1]. Domestic and wild species of felines act as the definitive hosts of the parasite, in which it undergoes both the sexual (gametogony) and asexual cycle (schizogony) [2]. Homeotherm species other than felids are known to act as the intermediate hosts in which the parasite can only undergo the asexual cycle [3,4].

There are two common routes of transmission between species. The first is through the ingestion of meat contaminated with tissue cysts, containing bradyzoites [3,5,6]. The second is through the ingestion of sporulated oocysts from vegetation, soil and water in the environment [5]. Vertical transmission, in which tachyzoites cross the placenta infecting the foetus and causing congenital toxoplasmosis, is a less frequent transmission route [6,7].

In sheep and goats, toxoplasmosis can cause abortions, resulting in economic losses for farmers [8,9,10]. In immune-competent hosts, *T. gondii* generally causes an asymptomatic infection, while hosts with a suppressed immune system are more susceptible to experiencing severe clinical manifestations from this parasite [11]. Recent publications, however, have indicated that immune-competent individuals are also able to experience a severe clinical disease and that the severity is possibly linked to the type of strain infecting the host [12]. Genetic analysis of isolates from around the world has revealed more diverse strains including a fourth clonal lineage, which can also possibly be linked to the severity of the infection [13,14,15]. Particularly in Central and South America, an abundance of atypical (non-clonal) strain types have been found, which may be due to a greater diversity and number of wild animal hosts occurring in these (sub-) tropical zones, each of which might favour the selection of different *T. gondii* genotypes, enabling a wider variety of strains to proliferate [16].

Research on toxoplasmosis in Africa is limited, with the majority of published material focusing on its incidence and prevalence in humans and livestock and little on wildlife. Until now, sampling in tropical regions has been done mainly on the American continent, so it is therefore necessary to understand the *T. gondii* population structures in other tropical regions such as Africa. The impact of *T. gondii* in wildlife species in Africa is poorly elucidated, including its clinical implications in wildlife species and especially in documenting the possible role it plays in the bridges found between human–livestock–wildlife interface areas. The excretion of oocysts from both domestic and wild felines dispersed in the environment possibly affect both herbivorous and omnivorous species found in an interface area. In many countries wildlife species are also a source of protein for many humans. These pathways are possible routes of transmission and a possible bridge that can be further investigated in interface areas. This is surprising as Africa contains a number of human–livestock–wildlife interface areas that co-exist and possibly affect each other. 

Therefore, the current review will systematically explore the past and current occurrence, prevalence and distribution of *T. gondii* in wildlife in Africa from its earliest mention to date. Additionally, the article will investigate the most commonly reported transmission routes for the different dietary wildlife types (herbivores, omnivores and carnivores). 

## 2. Results

### 2.1. Search Results

**Figure 1:**
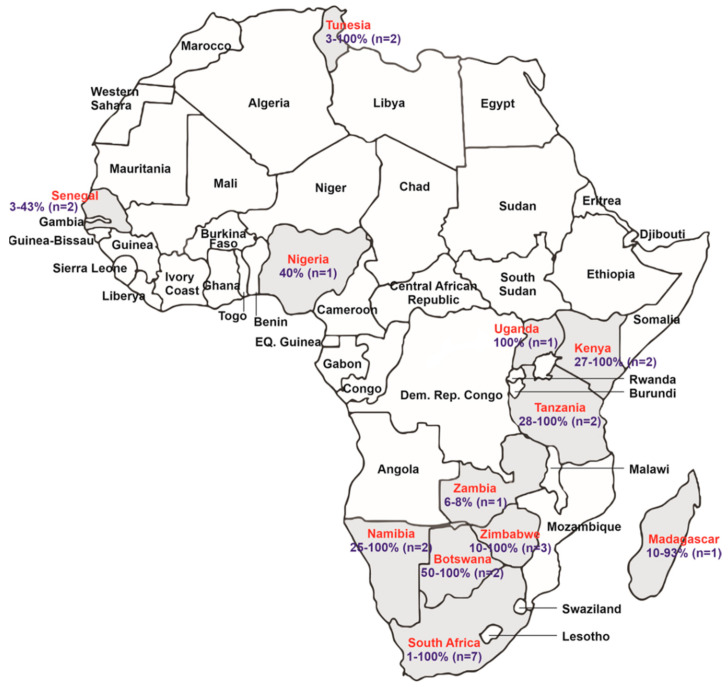
The African continent summarizing the prevalence ranges (%) and the number of studies done (*n*) in wildlife species in each documented country from the records included in the study. Only the African countries with published studies on *T. gondii* in wildlife species are highlighted in grey.

A total of 188 records were retrieved, 145 were found through a database search, whereas 43 additional records were identified through snowballing of reference lists of relevant reviews and research articles (PRISMA Flow diagram: Appendix B). After duplicate removal, the title/abstracts of 103 records were screened for relevance. Next, the full text articles of 86 remaining records were further evaluated against the inclusion and exclusion criteria. Twenty records were removed for not being in line with the objectives of this review. Finally, a total of 66 publications were retained and included in the qualitative synthesis. The quantitative synthesis included 20 articles with the majority of the records reporting data from South Africa (*n* = 7), followed by Zimbabwe (*n* = 3), Kenya (*n* = 2), Tanzania (*n* = 2), Botswana (*n* = 2), Namibia (*n* = 2), Uganda (*n* = 1), Zambia (*n* = 1), Nigeria (*n* = 1), Madagascar (*n* = 1), Senegal (*n* = 2) and Tunisia (*n* = 2) (Figure 1).

### 2.2. Historical Overview of T. gondii in African Wildlife

All prevalence data of *T. gondii* in African wildlife, as extracted from the retained records, are summarised and presented chronologically in Table 1. A summary that visually distinguishes between the countries with studies focusing on *T. gondii* in wildlife species (including the prevalence ranges) and the African countries that still need to be investigated are presented in Figure 1. 

The earliest mention of *T. gondii* in wildlife in Africa, was in a rodent (*Ctenodactylys gundi*) published by Nicolle and Manceaux in 1908 in Tunisia, northern Africa. The researchers were initially investigating leishmaniosis but instead detected tachyzoites and discovered *T. gondii* [17]. A few decades later, *T. gondii* was detected for the first time in a carnivore in Africa. In the Zoological Gardens in South Africa, Hofmeyr (1956) performed a necropsy on a cape hunting dog (*Lycaon pictus*, also known as African wild dog) and microscopically detected parasites that were identified as *T. gondii* [18] (Table 1). This finding raised the awareness of the possible dissemination of *T. gondii* in wildlife species in Africa and therefore prompted more surveys to be done in a wider diversity of wildlife species.

In 1975 scholars at the University of California noticed that a number of their imported African exotic animals tested positive for *T. gondii*. This led to an investigation to determine the seroprevalence in free-living wild animals from Tanzania, Uganda, Zambia and Kenya using an indirect hemagglutination test (IHA). Seropositivity was found in four African elephants (*Loxodonta Africana*, *n* = 63, 6%), one lion (*Panthera leo*, *n* = 1, 100%), two defessa waterboks (*Kobus ellipsiprymnus, n* = 2, 100%), eight Burchell zebras (*Equus quagga burchelli*, *n* = 29, 28%), one rock hyrax (*Procavia capensis*, *n* = 1, 100%) and four hippopotamuses (*Hippopotamus amphibious, n* = 49, 8%). This study documented the earliest serological detection of *T. gondii* specifically in a wild felid and in a variety of wild herbivores, omnivores and carnivores in Africa [19]. 

Subsequently, in 1980, a serological survey in Kenya detected *T. gondii* infection in free ranging herbivorous and carnivorous captive wild mammals. A Sabin–Feldman dye test (SFDT) was conducted, which resulted in seroprevalence ranges of 50–100% in the investigated carnivore and 27–100% in the included herbivore species, indicated in Table 1 [20].

In Nigeria, severe acute toxoplasmosis was documented in two lions (*P. leo*) from the Jos Zoological Gardens [21]. The two lions were kept in a cage with three other lions. The two lions had been experiencing severe symptoms such as depression of the respiratory system, dypsnoea, and excessive diarrhoea. One lion was treated with a combination of neomycin and methscopolamine while the other lion was treated with oxytetracycline. The condition of the two lions (*P. leo*) did not improve after treatment, and led to one dying after seven days of treatment and the other being euthanased after symptoms worsened [21]. An SFDT was taken on their blood samples before their death and was positive for *T. gondii*. The researchers found necrosis in the tissues and identified tachyzoites in the tissue cells. *Toxoplasma gondii*-like oocysts were also detected from their faecal material and this is possibly the earliest identification of oocysts in wild felids in Africa; however, the researchers never confirmed whether the oocysts were *T. gondii* oocysts [21]. Another study was done on lions (*P. leo*) from the Etosha National Park in Namibia. Serology was performed on 63 serum samples from free-ranging lions *(P. leo*) using the indirect fluorescent assay (IFA). The researchers found a high seroprevalence (96%) [22].

A high seroprevalence of 100% (*n* = 16) was also detected in African wild dogs (*Lycaon pictus*) in South Africa using an IFAT [23]. The researchers indicated that there was significant decline in the wild dog population and although this was attributed to a combination of factors such as loss of habitat, a decline in the availability of prey and inter- and intra-species competition, the burden of diseases was possibly another vital factor in the fall in population numbers [23]. Researchers from Auburn University (USA), also found a high seroprevalence of *T. gondii* in wild felids from the southern part of Africa [24]. The survey was done on a variety of free-ranging and captive felids in South Africa, Botswana and Namibia using an IFAT. The researchers were investigating the seroprevalence of both *Neospora caninum* and *T. gondii* in felids and found that *T. gondii* was more prevalent. The seroprevalence ranged from 56–100% in lions (*P. leo*), 33–50% in cheetahs (*Acinonyx jubatus*) and 50–100% in leopards (*Panthera pardus*) [24]. 

Penzhorn et al. (2002) also determined the seroprevalence of *T. gondii* in a variety of wild felids from South Africa, Botswana and Zimbabwe. The seroprevalence in lions was 100% (*n* = 42) in South Africa, 92% (*n* = 53) in Botswana and 100% (*n* = 21) in Zimbabwe. In leopards, the seroprevalence was 100% (*n* = 1) and 86% (*n* = 7) in Botswana and South Africa, respectively [25]. Using a modified agglutination test (MAT) on diverse carnivorous and herbivorous species, Hove and Mukaratirwa (2005) detected a seroprevalence of 92% (*n* = 26) in lions (*P. leo*), 10% (*n* = 10) in giraffe (*Giraffa camelopardalis*), 20% (*n* = 10) in greater kudu (*Tragelaphus strepsiceros*), 90% (*n* = 10) in nyala (*Tragelaphus angasii*), 57% (*n* = 14) in bushbucks (*Tragelaphus criptus*), 27% (*n* = 11) in black rhinos (*Diceros bicornis*) and 10% (*n* = 20) in African elephants (*Loxodonta africana*) [26]. Another study investigating the presence of *T. gondii* in a predominantly herbivorous species was conducted in Madagascar in black lemurs (*Eulemur macaco macaco*) [27]. By using a serum biological profile technique that detected both IgG and IgM antibodies against *T. gondii*, the researchers detected a prevalence of 10% (*n* = 10) [27]. 

A seroprevalence of 43% (*n* = 7) was also detected in captive lions (*P. leo*) in a zoo (Hann Dakar) in Senegal using an ELISA [28]. Another study, also focussing on captive lions, detected *Toxoplasma*-like oocysts in 17% (*n* = 30) of their population using a modified McMaster technique [29]. The researchers did stress the uncertainty regarding their identification of the parasite and therefore only reported their findings as *Toxoplasma*-like [29]. 

One of the endangered wildlife species native to Madagascar, the fossa (*Cryptoprocta ferox*) had a high seroprevalence of *T. gondii* at 93% (*n* = 45). The researchers indicated that the extinction of most of their carnivorous native species is, among other reasons, due to the burden of diseases and this could possibly be due to the increased infiltration of cats and dogs in the area which also affect the wildlife habitats [30]. 

A study was done in South Africa on brain samples from 106 free-ranging birds and four chickens, mostly from Limpopo and a few from the Free State, KwaZulu-Natal, Mpumalanga, Northern Cape and North-West provinces [31]. The brains were collected opportunistically from birds found deceased due to roadkill, turbulent weather, treatment failure, infections and unknown circumstances. Using a polymerase chain reaction assay (PCR), *T. gondii* was detected in three bird species with a prevalence of 25% (*n* = 4) in southern yellow-billed hornbills (*Tockus leucomelas*), 25% (*n* = 4) in laughing doves (*Spilopelia senegalensis*) and 20% (*n* = 5) in red-eyed doves (*Streptopelia semitorquata*), which were all from the Limpopo province [31]. The researchers further characterised the DNA extracted from positive birds using a single multiplex PCR with 15 microsatellite markers and only detected eight markers from the red-eyed doves which were specific to a type II strain. To our knowledge this is the only study that specifically focused on the detection of *T. gondii* in wild bird species in South Africa and on the genotyping in the whole of Africa in wildlife species [31]. 

Another study, also done by Lukášová et al. (2018), investigated the seroprevalence of *T. gondii* in a variety of herbivorous, omnivorous and carnivorous wildlife species in South Africa. An enzyme linked immunosorbent assay (ELISA) was used to determine the seroprevalence, which was 1% (*n* = 122), 8% (*n* = 13), 25% (*n* = 4) and 14% (*n* = 7) in gerbils (*Gerbilliscus* sp.), kudus (*Tragelaphus strepsiceros*), honey badgers (*Mellivora capensis*) and white-tailed mongooses (*Ichneumia albicauda*), respectively [32]. Antibodies to *T. gondii* were also detected in 60 of 80 (75%) spotted hyenas (*Crocuta crocuta*) in Tanzania using an ELISA [33]. A prevalence study was done on captured wild rodents and shrew in Senegal using serology (MAT) and a molecular analysis (PCR). Seroprevalence results were 4.8% (*n* = 671), 2.6% (*n* = 78), 31.9% (*n* = 47) and 37.5% (*n* = 32) in *Mus musculus domesticus* (rodent species), *Rattus*, *Cricetomys gambianus* (rodent species) and *Crocidura olivieri* (shrew), respectively [34]. The PCR results of each of the rodent species are presented in Table 1 [34]. 

Another rare survey was done on yellow-legged gulls (*Larus michahellis*) in Tunisia. The researchers performed serology on sampled eggs to avoid the logistical challenges of capturing wild birds [35]. The researchers further indicated that by sampling the eggs this could be a more efficient way to measure the exposure of the females breeding in the area [35]. The eggs were collected from two locations, namely Sfax and Djerba, with 3% and 11% of seroprevalence determined, respectively, using an ELISA [35]. 

A high overall seroprevalence of 83% (*n* = 29) was detected in caracals (*Caracal caracal*) in South Africa using an IFAT that serologically detected both IgM and IgG anti-*T. gondii* antibodies [36]. A seroprevalence of 79% (*n* = 29) and 38% (*n* = 29) was also determined using an IFAT that separately detected IgG and IgM, respectively [36]. 

Lastly, free-ranging wildlife species were surveyed in Namibia using an ELISA and further confirmed using an immunoblot technique (IB). The carnivore species investigated were African lions (*P. leo*), brown hyenas (*Hyaena brunnea*), caracals (*Caracal caracal*), cheetahs (*A. jubatus*), leopards (*P. pardus*), spotted hyena (*Crocuta crocuta*), African wild dogs (*L. pictus*), bat eared foxes (*Otocyon megalotis*) and black-backed jackals (*Canis mesomelas*) with seroprevalence estimates ranging between 25 and 93% [37]. In blue wildebeests (*Connochaetes taurinus*), the seroprevalence of *T. gondii* was 10% [37].

## 3. Discussion

The information obtained from this systematic review indicates that *T. gondii* is prevalent and widespread in wildlife in Africa. Overall, however, the number of studies published on the topic is quite limited and the majority of records are focused on samples from countries with well-developed wildlife reserves; similarly, few of these records report data from samples obtained in areas at the human–livestock–wildlife interface. Furthermore, data were only available for twelve countries on the African continent, leaving many blind spots on the map for the distribution of *T. gondii* in wildlife in Africa.

The prevalence estimates, obtained by a wide range of techniques, were highly variable, ranging between 0 and 100%. Aside from the records with a positive detection of *T. gondii* identified in our systematic review, few other studies have investigated the presence of *T. gondii* in wildlife in Africa but failed to detect the parasite or only indicated protozoan parasite detection but with no *T. gondii* confirmation in predominantly herbivorous and omnivorous wild species from Madagascar, Kenya, Tanzania and Uganda [19,20,38,39,40,41]. 

Overall, the prevalence estimates reported in the records identified in the current systematic review, suggest that *T. gondii* seems to be more prevalent in carnivores compared to herbivores [26,27,39], which is consistent with several studies in wildlife in USA and Europe. For instance, Marchiondo et al. (1976) in the USA found a higher seroprevalence in carnivores (45%) compared to omnivores (28%) and herbivores (21%) [42]. Another study in the USA also found a higher prevalence in carnivores (66%) when compared to omnivores (11%) and herbivores (15%) [43]. Similarly, in Europe, researchers also found a higher seroprevalence in carnivores (20.21%) and omnivores (16.91%) when compared to herbivores (0–2.48%) [4]. From these data we can assume that the most common route of transmission in wild carnivores is through the ingestion of infected meat [43]. This could also be due to the fact that carnivores are higher on the food pyramid. Unfortunately, information on the prevalence of *T. gondii* in potential prey such as herbivores and omnivores is even more limited in Africa, hampering more accurate deductions. Further research targeted at wildlife with association to the different feeding types is required [12,26,27]. 

Severe toxoplasmosis has been described in a few studies, similar to the two cases mentioned in the two lions and the cape hunting dog (African wild dog) [18,21]. In the USA, Dubey (1987) and Smith et al. (1995) described severe clinical toxoplasmosis in captive bobcats (*Lynx rufus*). Smith et al. (1995) found necrosis in the liver, renal pelvis, heart and skeletal muscle tissue of a sick bobcat *(L. rufus*) that was serologically positive for *T. gondii* [44]. The bobcat documented by Dubey (1987) had died only one week after birth. In another clinical case reported in the USA, a sick wild turkey (*Meleagris gallopavo*) died shortly after being captured. Necrosis was detected on the kidneys, liver, spleen and pulmonary interstitium and toxoplasmosis was confirmed using an avidin-biotin immunohistochemical technique in liver sections [45]. Data on the clinical impact of toxoplasmosis in wildlife species is limited in Africa and should further be investigated. 

In the two cases of the severely infected lions, Ocholi et al. (1989) further states that the possible reason why the remaining lions living in the same cage did not experience a clinical disease even though they were serologically positive for *T. gondii*, could have been due to a number of factors such as the type of strain of the parasite, the age of the animals, the organs specifically infected and the animals’ immunological response [21]. Due to the evidence of strain diversity of *T. gondii*, researchers have indicated concerns regarding the discovery of more diverse strains resulting in possible severe infections in hosts [13,14]. A study undertaken in the United Kingdom screened wildlife species including, ferrets (*Mustela putorius furo*), red foxes (*Vulpes vulpes*), polecats (*Mustela putorius*), minks (*Neovison vison*), badgers (*Meles meles*) and stoats (*Mustela erminea*) using polymerase chain reaction—restriction fragment length polymorphism (PCR-RFLP) with satellite markers. The researchers found all three clonal genotypes I, II and III with genotype II being dominant [46]. In a study in the USA in otters (*Enhydra lutris*), mountain lions (*Puma concolor*) and foxes (*Vulpes vulpes*), the common three clonal lineages were also found by PCR and DNA sequencing, but additionally a diverse strain referred to as Genotype X was identified [46]. In Brazil, a high diversity of non-clonal strains was found by PCR-RFLP in wild Felidae, including, jaguarundi (*Puma yagouaroundi*), Geoffroy’s cat (*Leopardus geoffroyi*), oncilla *(L. tigrinus*), margay (*L. wiedii*), ocelot (*L. pardalis*) and pampas cat (*L. colocolo*). Two new genotypes, Lw#31Tn and Py#21Sm and one previously described genotype Py#56Br were identified [47]. Currently, there is very limited data on the *T. gondii* strains circulating in African wildlife [16].

The case documented by Dubey (1987) in bobcat kittens, of which two died within the first week after birth indicates the possibility of congenital infection [48]. Similar cases of congenital toxoplasmosis have been documented in white-tailed deer (*Odocoileus virginianus*) and pallas cats (*Otocolobus manul*) from USA and Austria, respectively [49,50]. These cases suggest the possible occurrence of congenital toxoplasmosis in wildlife species in Africa [48]. Congenital toxoplasmosis is rarely documented in wildlife worldwide including the possibility of *T. gondii* related abortions in wildlife species.

A high prevalence of *T. gondii* in humans and livestock is assumed to be associated with the presence of cats. *Toxoplasma gondii* only occurs where felids are present [22,24]. Publications have mentioned that a high prevalence of this parasite in wild herbivores indicates that the most common mode of transmission is through contamination of the environment with sporulated oocysts, wild felids being the shedders [46,51,52]. To our knowledge there has been only one publication in Africa which documents the detection of oocysts in an African lion (*P. leo*). However, in that case no techniques were used to differentiate *T. gondii* oocysts from those of *Hammondia hammondi*, a non-zoonotic coccidian protozoa of felids, of which the oocysts bare a close morphological resemblance to those of *T. gondii* [21,51]. In contrast to Africa, there are numerous studies in countries such as the Czech Republic, Belgium and the USA showing oocyst production in wild felids. Oocyst identification has been done in a variety of wild felids found globally which are confirmed definitive hosts, these include bobcat (*L. rufus*), cheetah (*A. jubatus*), mountain lion (*Puma concolor*), wild cat (*Felis silvestris*), Siberian tiger (*Panthera tigris altaica*), amur leopard cat (*Panthera pardus orientalis*), Geoffroy’s cat (*L. geoffroyi*), cougar (*Felis concolor*), pallas cat (*F. manul*), jaguarundi (*Puma yagouaroundi*) and ocelot (*Felis pardalis*) [42,50,52,53,54,55]. There are still a number of wild felid species in Africa that have not yet been surveyed for *T. gondii* prevalence or oocyst shedding such as serval (*Leptailurus serval*), caracal (*Caracal caracal*), African wildcat (*Felis silvestris lybica*), African golden cat (*Caracal aurata*), jungle cat (*Felis chaus*) and the black-footed cat (*Felis nigripes*). Although this is the case, confirmed reports of oocyst shedding from wild felids found worldwide can lead us to speculate that the unconfirmed wild felids found in Africa play a similar role as definitive hosts and are possibly essential in the dissemination and preservation of the parasite in the different ecological niches forming a possible bridge where human dwellings, livestock and wildlife interface.

Strong winds and rainfall can disperse oocysts produced by both domestic and wild felids and can cause contamination of the environment across habitats whether fenced or unfenced [56]. Smaller animals such as rodents can also act as reservoirs of the parasite, since, due to their size, they can easily move through habitats and possibly disseminate the parasite within the different communities. Lastly, in numerous countries humans are known to hunt wild mammals and birds for consumption and this increases the probability of human infection [4]. This shows that the circulation of *T. gondii* in wildlife can possibly have an effect on human and livestock populations living in close proximity and vice versa, with the transfer of pathogens between habitats, especially zoonotic pathogens, being a potentially primary concern [2,30].

The reviewed publications included in the study used a variety of direct and indirect *T. gondii* detection methods. The direct methods included in the studies are microscopy and PCR techniques [57]. Microscopic detection includes the examination of faeces, water and environmental samples for oocysts and tissue samples for tachyzoites and tissue cysts. Although microscopy of tissue samples is considered specific it is a subjective technique (with potential misinterpretation of results) that can also be unreliable. It also lacks sensitivity especially when using light microscopy, but can be improved by staining (Giemsa, Haematoxylin and Eosin, and the Periodic acid Schiff) or immunofluorescent or immunohistological staining [57,58]. 

Molecular techniques (PCR) such nested-PCR (nPCR), multiplex PCR and quantitative-PCR (qPCR) can be highly sensitive and specific as they directly target the parasite DNA [58,59]. The analytical sensitivity of nPCR techniques range from 96–99% and the specificity ranges between 95–100%, both in blood, urine and foetal tissues [59,60]. These techniques use DNA extracted from various samples and can be further utilised for DNA characterisation and genotyping (excluding qPCR) [57,58].

Serological techniques are commonly used when determining the prevalence in hosts and this is because *T. gondii* evokes a very strong and long lasting immunological response in hosts [59]. The assays use blood or constituents of blood (serum and plasma) for the detection of anti-*T. gondii* antibodies (IgG, IgM and IgA), which can also help in distinguishing between acute and chronic infections. The most frequently used serological tests in the reviewed articles are the Sabin–Feldman dye test (SFDT), enzyme-linked immunosorbent assay (ELISA) and modified agglutination test (MAT). The SFDT is considered the gold standard serological technique. It has been reported to be both highly sensitive and specific. However, it requires the culturing of live parasites in mice or tissue cultures making it very technical and hazardous [58]. In sows, the sensitivity of ELISA, indirect haemagglutination assay (IHA), MAT, latex agglutination test (LAT) and SFDT was estimated at 72.9%, 29.4%, 82.9%, 45.9%, and 54.4%, respectively, while the specificities were estimated at 85.9%, 98.3%, 90.3%, 96.9% and 90.8%, respectively [61]. Another serological study undertaken in sheep found high sensitivities of 96%, 90.1% and 80.4% using MAT, ELISA and indirect fluorescent assay (IFAT), respectively. The detected specificities were 88.9% using MAT, 85.9% using ELISA and 91.4% using IFAT. Due to the possibility of false negative reactions on serological assays, it is advisable to perform more than one detection assay, as is reported in some studies [23,36,39]. 

Serological prevalence studies are more common than molecular studies on *T. gondii* in wildlife species. Only few studies have investigated the specificity and sensitivity of the different detection techniques and validated them for use in wildlife species due to the difficulty in accessing wildlife samples [62]. Thus, highlighting another gap in *Toxoplasma* research in wildlife. 

Our study has some limitations. Most records retrieved in this systematic review report the findings of studies applying convenience sampling at small sizes. Therefore, the reported prevalence estimates might not be representative for the entire population of each of the investigated wildlife species due to selection bias. For instance, in domestic animals (and humans), it is known that seroprevalence increases with age as a result of longer exposure [63]. Moreover, the small sample sizes will inherently lead to imprecise estimates. Finally, the identified records used a wide range of, mostly serological, techniques to detect the presence of *T. gondii* in wildlife, many of which have not been validated for use in the investigated wildlife species. 

## 4. Materials and Methods

### 4.1. Search Strategy

The aim of the study was to summarize existing knowledge on the occurrence, prevalence, distribution and history of *T. gondii* in wildlife on the African continent. PRISMA guidelines were used for reporting the review process [64] (PRISMA checklist: Appendix B). Relevant records, published between 1 January 1900 and 31 December 2020, were searched by means of three international bibliographic databases: PubMed (https://pubmed.ncbi.nlm.nih.gov/, accessed on 3 February 2021), Web of Science (https://webofknowledge.com, accessed on 3 February 2021) and CAB Direct (https://cabdirect.org, accessed on 3 February 2021) (Protocol: Appendix A). A search phrase was developed for use in the bibliographic databases, based in part on the phrase developed by Pienaar et al. [65]: (*Toxoplasma gondii* OR Toxoplasmosis OR *T. gondii*) AND (zoo OR wildlife OR wild) AND ((Africa OR African continent OR Africa OR Algeria OR Angola OR Benin OR Botswana OR Burkina Faso OR Burundi OR Cameroon OR Canary Islands OR Cape Verde OR Central African Republic OR Chad OR Comoros OR Congo OR Democratic Republic of Congo OR Djibouti OR Egypt OR Equatorial Guinea OR Eritrea OR Ethiopia OR Gabon OR Gambia OR Ghana OR Guinea OR Guinea Bissau OR Ivory Coast OR Cote d’Ivoire OR Jamahiriya OR Jamahiryia OR Kenya OR Lesotho OR Liberia OR Libya OR Libia OR Madagascar OR Malawi OR Mali OR Mauritania OR Mauritius OR Mayote OR Morocco OR Mozambique OR Mocambique OR Namibia OR Niger OR Nigeria OR Principe OR Reunion OR Rwanda OR Sao Tome OR Senegal OR Seychelles OR Sierra Leone OR Somalia OR South Africa OR St Helena OR Sudan OR Swaziland OR Tanzania OR Togo OR Tunisia OR Uganda OR Western Sahara OR Zaire OR Zambia OR Zimbabwe OR Central Africa OR Central African OR West Africa OR West African OR Western Africa OR Western African OR East Africa OR East African OR Eastern Africa OR Eastern African OR North Africa OR North African OR Northern Africa OR Northern African OR South African OR Southern Africa OR Southern African OR sub Saharan Africa OR sub Saharan African OR subSaharan Africa OR subSaharan African) NOT (guinea pig OR guinea pigs OR aspergillus niger)). Furthermore, reference lists of retained records and/or review articles were snowballed for relevant sources.

### 4.2. Selection Criteria

After extracting the records from the three databases, duplicate records were re-moved, and the titles and abstracts were screened for relevance. The inclusion criteria included studies reporting data on *T. gondii* from the African continent in both free ranging and captive wild species. The exclusion criteria were: (i) publications on parasites other than *T. gondii*, (ii) records documenting the detection of *T. gondii* in domestic species rather than wildlife species, (iii) studies reporting/using data older than 1900 or published after 31 December 2020, (v) studies that were conducted in countries outside the African continent, (vi) publications with information not in line with the review question (prevalence, detection and history of *T. gondii* in African wildlife), and lastly, (vi) duplicate studies. Subsequent to the screening process, full texts were evaluated using the same criteria described above. 

### 4.3. Data Extraction and Analysis

The following variables were extracted from the articles and entered into Microsoft Excel worksheets: author name and publication year, country, species name, reported prevalence, method of detection.

## 5. Conclusions

The current review highlights a substantial gap on the research done on *T. gondii* in wildlife in Africa. The lack of knowledge in Africa, particularly in areas where the human–livestock–wildlife overlap, prevents us from determining its impact and distribution in the different habitats. This lack also prevents us from determining the specific role played by the wild cycle and possibly the direct or indirect implications it might have on the public health of the surrounding habitats and the occupants affected, knowledge which would aid the achievement of better disease control, diagnosis and treatment. It is also important to investigate the common circulating genotypes, whether there is evidence of genetic variation, and the possibility of congenital toxoplasmosis in order to better understand the parasite and the severity of the clinical infection experienced by the hosts. It is therefore important to undertake further research in these areas. 

## Figures and Tables

**Table 1:** *T. gondii* detection in wildlife species in Africa.

Country	Common Animal Species Name	Scientific Name	Prevalence% (Positive/*n*)	Methods of Detection	Reference
Tunisia	Common gundi ^H^	*Ctenodactylys gundi*	100 (3/3)	PM, MC	Nicolle and Manceaux, 1908
South Africa	African wild dog ^C^	*Lycaon pictus*	50 (1/2)	MC	Hofmeyr, 1956
Kenya	Lion ^C^	*Panthera leo*	100 (1/1)	IHA	Riemann et al., 1975
Uganda	Defassa waterbuck ^C^	*Kobus ellipsiprymnus*	100 (2/2)	IHA	Riemann et al., 1975
Tanzania	Rock Hyrax ^O^	*Procavia capensis*	100 (1/1)	IHA	Riemann et al., 1975
Tanzania	Burchells Zebra ^H^	*Equus quagga burchellii*	28 (8/29)	IHA	Riemann et al., 1975
Zambia	African elephant ^H^	*Loxodonta africana*	6 (4/63)	IHA	Riemann et al., 1975
Zambia	Hippopotamus ^H^	*Hippopotamus amphibius*	8 (4/49)	IHA	Riemann et al., 1975
Kenya	Silver-backed jackal ^C^	*Canis mesomelas*	67 (4/6)	SFTD	Bakal et al., 1980
Kenya	White tailed mongoose ^C^	*Ichneumia albicauda*	50 (1/2)	SFTD	Bakal et al., 1980
Kenya	Spotted hyena ^C^	*Crocuta crocuta*	100 (6/6)	SFTD	Bakal et al., 1980
Kenya	Zebra ^H^	*Equus quagga burchellii*	90 (9/10)	SFTD	Bakal et al., 1980
Kenya	Warthog ^O^	*Phacochoerus africanus*	100 (2/2)	SFTD	Bakal et al., 1980
Kenya	Giraffe ^H^	*Giraffa camelopardalis*	50 (5/10)	SFTD	Bakal et al., 1980
Kenya	Eland ^H^	*Taurotragus oryx*	100 (10/10)	SFTD	Bakal et al., 1980
Kenya	Bushbuck ^H^	*Tragelaphus scriptus*	80 (8/10)	SFTD	Bakal et al., 1980
Kenya	Fringe-eared oryx ^H^	*Oryx beisa callotis*	50 (2/4)	SFTD	Bakal et al., 1980
Kenya	Waterbuck ^H^	*Kobus ellipsiprymnus*	27 (5/11)	SFTD	Bakal et al., 1980
Kenya	Hartebeest ^H^	*Alcelaphus buselaphus*	83 (10/12)	SFTD	Bakal et al., 1980
Kenya	Topi ^H^	*Damaliscus lunatus jimela*	82 (9/11)	SFTD	Bakal et al., 1980
Kenya	Wildebeest ^H^	*Connochaetes taurinus*	90 (9/10)	SFTD	Bakal et al., 1980
Kenya	Impala ^H^	*Aepyceros melampus*	80 (8/10)	SFTD	Bakal et al., 1980
Kenya	Grant’s gazelle ^H^	*Nanger granti*	80 (8/10)	SFTD	Bakal et al., 1980
Kenya	Thomson’s gazelle ^H^	*Eudorcas thomsonii*	90 (9/10)	SFTD	Bakal et al., 1980
Kenya	Steenbok ^H^	*Raphicerus campestris*	50 (1/2)	SFTD	Bakal et al., 1980
Kenya	Dikdik ^H^	*Rhynchotragus* spp.	100 (5/5)	SFTD	Bakal et al., 1980
Kenya	African buffalo ^H^	*Syncerus caffer*	100 (10/10)	SFTD	Bakal et al., 1980
Nigeria	Lion ^C^	*Panthera leo*	40 (2/5)	SFTD, PM, MT, MC oocysts in feces	Ocholi et al., 1989
South Africa	Lion ^C^	*Panthera leo*	90 (36/40)	IFAT	Cheadle et al., 1999
South Africa	Leopard ^C^	*Panthera pardus*	100 (2/2)	IFAT	Cheadle et al., 1999
Botswana	Leopard ^C^	*Panthera pardus*	50 (1/2)	IFAT	Cheadle et al., 1999
Namibia	Lion ^C^	*Panthera leo*	100 (1/1)	IFAT	Cheadle et al., 1999
Namibia	Cheetah ^C^	*Acinonyx jubatus*	33 (2/6)	IFAT	Cheadle et al., 1999
South Africa	Cheetah ^C^	*Acinonyx jubatus*	50 (8/16)	IFAT	Cheadle et al., 1999
South Africa	African wild dog ^C^	*Lycaon pictus*	100 (16/16)	IFAT	Van Heerden et al., 1993
Botswana	Lion ^C^	*Panthera leo*	92 (49/53)	IFAT	Penzhorn et al., 2002
Botswana	Leopard ^C^	*Panthera pardus*	100 (1/1)	IFAT	Penzhorn et al., 2002
South Africa	Lion ^C^	*Panthera leo*	100 (42/42)	IFAT	Penzhorn et al., 2002
South Africa	Leopard ^C^	*Panthera pardus*	86 (6/7)	IFAT	Penzhorn et al., 2002
Zimbabwe	Lion ^C^	*Panthera leo*	100 (21/21)	IFAT	Penzhorn et al., 2002
Zimbabwe	Giraffe ^H^	*Giraffa camelopardalis*	10 (1/10)	MAT	Hove and Mukaratirwa, 2005
Zimbabwe	Greater kudu ^H^	*Tragelaphus strepsiceros*	20 (2/10)	MAT	Hove and Mukaratirwa, 2005
Zimbabwe	Nyala ^H^	*Tragelaphus angasii*	90 (9/10)	MAT	Hove and Mukaratirwa, 2005
Zimbabwe	Bushbuck ^H^	*Tragelaphus scriptus*	57 (8/14)	MAT	Hove and Mukaratirwa, 2005
Zimbabwe	Black rhino ^H^	*Diceros bicornis*	27 (3/11)	MAT	Hove and Mukaratirwa, 2005
Zimbabwe	African elephant ^H^	*Loxodonta africana*	10 (2/20)	MAT	Hove and Mukaratirwa, 2005
Zimbabwe	Lion ^C^	*Panthera leo*	92 (24/26)	MAT	Hove and Mukaratirwa, 2005
Zimbabwe	Ostrich ^H^	*Struthio camelus*	48 (24/50)	MAT	Hove and Mukaratirwa, 2005
Madagascar	Black lemur ^H^	*Eulemur macaco*	10 (1/10)	Serum biochemical profile (IgG and IgM)	Junge et al., 2007
Senegal	Lion ^C^	*Panthera leo*	43 (3/7)	ELISA	Kamga-Waladjo et al., 2009
Zimbabwe	Lion ^C^	*Panthera leo*	17 (5/30)	McMaster (feces)	Makarati et al., 2013
Madagascar	Fossa ^C^	*Cryptoprocta ferox*	93 (42/25)	ELISA	Pomerantz et al., 2016
South Africa	Gerbil ^H^	*Gerbilliscus* sp.	1 (1/122)	ELISA	Lukášová et al., 2018
South Africa	Kudu ^H^	*Tragelaphus strepsiceros*	8 (1/13)	ELISA	Lukášová et al., 2018
South Africa	Honey badger ^C^	*Mellivora capensis*	25 (1/4)	ELISA	Lukášová et al., 2018
South Africa	White tailed mongoose ^C^	*Ichneumia albicauda*	14 (1/7)	ELISA	Lukášová et al., 2018
South Africa	Southern Yellow-billed Hornbill (bird) ^O^	*Tockus leucomelas*	25 (1/4)	PCR (brain)	Lukášová et al., 2018
South Africa	Laughing Dove (bird) ^O^	*Spilopelia senegalensis*	25 (1/4)	PCR (brain)	Lukášová et al., 2018
South Africa	Red-eyed Dove (bird) ^O^	*Streptopelia semitorquata*	20 (1/5)	PCR (brain)	Lukášová et al., 2018
Tanzania	Spotted hyena ^C^	*Crocuta*	75 (45/60)	ELISA	Ferreira et al., 2018
Senegal	Rodents ^O^	*Mus musculus domesticus*	4.8 (32/671) and 13.1 (88/671)	MAT and PCR	Galal et al., 2019
Senegal	Rodents ^O^	*Rattus rattus*	2.6 (2/78) and 3.8 (3/78)	MAT and PCR	Galal et al., 2019
Senegal	Rodents ^O^	*Cricetomys gambianus*	31.9 (15/47) and 27.7 (13/47)	MAT and PCR	Galal et al., 2019
Senegal	Shrew ^O^	*Crocidura olivieri*	37.5 (12/32) and 15.6 (5/32)	MAT and PCR	Galal et al., 2019
Tunisia	Yellow-legged gull ^O^	*Larus michahellis*	3 (30 nests, Sfax), 11 (37 nest, Djerba)	ELISA	Gamble et al., 2019
South Africa	Caracal ^C^	*Caracal*	83 (24/29)	IFAT	Serleys et al., 2019
Namibia	Blue wildebeest ^H^	*Connochaetes taurinus*	10 (2/20) and	ELISA and IB	Seltmann et al., 2020
Namibia	Honey badger ^C^	*Mellivora capensis*	70 (7/10) and 60 (6/10)	ELISA and IB	Seltmann et al., 2020
Namibia	Lion ^C^	*Panthera leo*	93 (55/59) and 93 (55/59)	ELISA and IB	Seltmann et al., 2020
Namibia	Brown Hyena ^C^	*Hyaena brunnea*	92 (12/13) and 92 (12/13)	ELISA and IB	Seltmann et al., 2020
Namibia	Caracal ^C^	*Caracal*	67 (10/15) and 67 (10/15)	ELISA and IB	Seltmann et al., 2020
Namibia	Cheetah ^C^	*Acinonyx jubatus*	52 (131/250) and 52 (131/250)	ELISA and IB	Seltmann et al., 2020
Namibia	Leopard ^C^	*Panthera pardus*	81 (47/58) and 81 (47/58)	ELISA and IB	Seltmann et al., 2020
Namibia	Spotted hyena ^C^	*Crocuta*	91 (10/11) and 91 (10/11)	ELISA and IB	Seltmann et al., 2020
Namibia	Wild dog ^C^	*Lycaon pictus*	71 (5/7) and 57 (4/7)	ELISA and IB	Seltmann et al., 2020
Namibia	Bat-eared fox ^O^	*Otocyon megalotis*	25 (1/4) and 0 (0/4)	ELISA and IB	Seltmann et al., 2020
Namibia	Black-backed jackal ^C^	*Canis mesomelas*	67 (26/39) and 67 (26/39)	ELISA and IB	Seltmann et al., 2020

^1^ Detection methods; IHA: indirect haemagglutination test; SFDT: Sabin–Feldman dye test; IFAT: indirect fluorescent antibody technique; ELISA: enzyme linked immunosorbent assay; PCR: polymerase chain reaction; MC: microscopy; PM: post-mortem assessment; MT: microtiter test; McMaster: modified McMaster technique. Dietary types; ^H^: herbivores; ^O^: omnivores; ^C^: carnivores.

**Figure A1:**
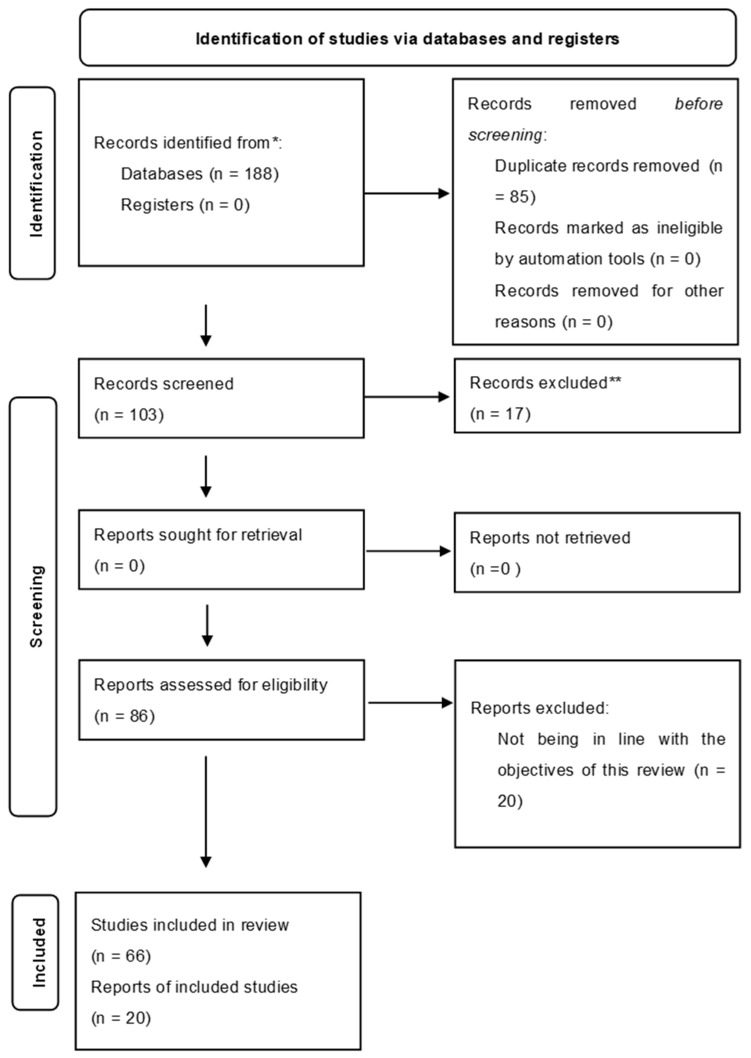
PRISMA 2020 Flow diagram. From: The PRISMA flow diagram was adapted from a diagram published by researcher Page et al. (2020) [66]. * *p* < 0.05, ** *p* < 0.01.

## Data Availability

The data generated and analysed during the current study are available in the manuscript in Appendix A and Appendix B.

## Appendix A

Supplementary documents

Systematic Review Protocol

Aim: The aim was to summarize knowledge on the presence of *Toxoplasma gondii* in wildlife in Africa.

Research questions:

- Which African countries have reported the presence of *T. gondii* in wildlife?
- What is the reported prevalence range of *T. gondii* in wildlife in Africa?
- What is the history of *T. gondii* in relation to its presence in wildlife in Africa?

Methods: The systematic review followed the Preferred Reporting Items for Systematic Reviews and Meta-Analyses (PRISMA) guidelines for reporting systematic reviews [64]. Briefly, records were extracted from the different sources, duplicates were removed, and title/abstract was screened for fitting the topic of the review. Next, the full texts of the retained articles were evaluated for eligibility and data were extracted for the final set of included records.

Sources:

- Bibliographic databases: Pubmed (https://pubmed.ncbi.nlm.nih.gov/, accessed on 3 February 2021), Web of Science (https://webofknowledge.com, accessed on 3 February 2021) and CAB Direct (https://cabdirect.org, accessed on 3 February 2021).
- Additional sources: reference lists of retained records and/or review articles were snowballed for relevant sources.

Search phrase:

(*Toxoplasma gondii* OR Toxoplasmosis OR *T. gondii*) AND (zoo OR wildlife OR wild) AND ((Africa OR African continent OR Africa OR Algeria OR Angola OR Benin OR Botswana OR Burkina Faso OR Burundi OR Cameroon OR Canary Islands OR Cape Verde OR Central African Republic OR Chad OR Comoros OR Congo OR Democratic Republic of Congo OR Djibouti OR Egypt OR Equatorial Guinea OR Eritrea OR Ethiopia OR Gabon OR Gambia OR Ghana OR Guinea OR Guinea Bissau OR Ivory Coast OR Cote d’Ivoire OR Jamahiriya OR Jamahiryia OR Kenya OR Lesotho OR Liberia OR Libya OR Libia OR Madagascar OR Malawi OR Mali OR Mauritania OR Mauritius OR Mayote OR Morocco OR Mozambique OR Mocambique OR Namibia OR Niger OR Nigeria OR Principe OR Reunion OR Rwanda OR Sao Tome OR Senegal OR Seychelles OR Sierra Leone OR Somalia OR South Africa OR St Helena OR Sudan OR Swaziland OR Tanzania OR Togo OR Tunisia OR Uganda OR Western Sahara OR Zaire OR Zambia OR Zimbabwe OR Central Africa OR Central African OR West Africa OR West African OR Western Africa OR Western African OR East Africa OR East African OR Eastern Africa OR Eastern African OR North Africa OR North African OR Northern Africa OR Northern African OR South African OR Southern Africa OR Southern African OR sub Saharan Africa OR sub Saharan African OR subSaharan Africa OR subSaharan African) NOT (guinea pig OR guinea pigs OR aspergillus niger)).

Note: for the African countries, we used the search phrase developed by Pienaar et al. (2011)

Search phrase translated for use in PubMed: (“*Toxoplasma gondii*” OR Toxoplasmosis OR “*T. gondii*”) AND (“animals, zoo”[MeSH] OR “animals, wild”[MeSH] OR Wildlife OR wild) AND ((Africa OR “African continent” OR “Africa”[MeSH] OR Africa*[tw] OR Algeria[tw] OR Angola[tw] OR Benin[tw] OR Botswana[tw] OR “Burkina Faso”[tw] OR Burundi[tw] OR Cameroon[tw] OR “Canary Islands”[tw] OR “Cape Verde”[tw] OR “Central African Republic”[tw] OR Chad[tw] OR Comoros[tw] OR Congo[tw] OR “Democratic Republic of Congo”[tw] OR Djibouti[tw] OR Egypt[tw] OR “Equatorial Guinea”[tw] OR Eritrea[tw] OR Ethiopia[tw] OR Gabon[tw] OR Gambia[tw] OR Ghana[tw] OR Guinea[tw] OR “Guinea Bissau”[tw] OR “Ivory Coast”[tw] OR “Cote d’Ivoire”[tw] OR Jamahiriya[tw] OR Jamahiryia[tw] OR Kenya[tw] OR Lesotho[tw] OR Liberia[tw] OR Libya[tw] OR Libia[tw] OR Madagascar[tw] OR Malawi[tw] OR Mali[tw] OR Mauritania[tw] OR Mauritius[tw] OR Mayote[tw] OR Morocco[tw] OR Mozambique[tw] OR Mocambique[tw] OR Namibia[tw] OR Niger[tw] OR Nigeria[tw] OR Principe[tw] OR Reunion[tw] OR Rwanda[tw] OR “Sao Tome”[tw] OR Senegal[tw] OR Seychelles[tw] OR “Sierra Leone”[tw] OR Somalia[tw] OR “South Africa”[tw] OR “St Helena”[tw] OR Sudan[tw] OR Swaziland[tw] OR Tanzania[tw] OR Togo[tw] OR Tunisia[tw] OR Uganda[tw] OR “Western Sahara”[tw] OR Zaire[tw] OR Zambia[tw] OR Zimbabwe[tw] OR “Central Africa”[tw] OR “Central African”[tw] OR “West Africa”[tw] OR “West African”[tw] OR “Western Africa”[tw] OR “Western African”[tw] OR “East Africa”[tw] OR “East African”[tw] OR “Eastern Africa”[tw] OR “Eastern African”[tw] OR “North Africa”[tw] OR “North African”[tw] OR “Northern Africa”[tw] OR “Northern African”[tw] OR “South African”[tw] OR “Southern Africa”[tw] OR “Southern African”[tw] OR “sub Saharan Africa”[tw] OR “sub Saharan African”[tw] OR “subSaharan Africa”[tw] OR “subSaharan African”[tw]) NOT (“guinea pig”[tw] OR “guinea pigs”[tw] OR “aspergillus niger”[tw])).

Search phrase translated for use in Web of Science and CAB Direct: (“*Toxoplasma gondii*” OR Toxoplasmosis OR “*T. gondii*”) AND (zoo OR wildlife OR wild) AND ((Africa OR “African continent” OR Africa* OR Algeria OR Angola OR Benin OR Botswana OR “Burkina Faso” OR Burundi OR Cameroon OR “Canary Islands” OR “Cape Verde” OR “Central African Republic” OR Chad OR Comoros OR Congo OR “Democratic Republic of Congo” OR Djibouti OR Egypt OR “Equatorial Guinea” OR Eritrea OR Ethiopia OR Gabon OR Gambia OR Ghana OR Guinea OR “Guinea Bissau” OR “Ivory Coast” OR “Cote d’Ivoire” OR Jamahiriya OR Jamahiryia OR Kenya OR Lesotho OR Liberia OR Libya OR Libia OR Madagascar OR Malawi OR Mali OR Mauritania OR Mauritius OR Mayote OR Morocco OR Mozambique OR Mocambique OR Namibia OR Niger OR Nigeria OR Principe OR Reunion OR Rwanda OR Sao Tome OR Senegal OR Seychelles OR “Sierra Leone” OR Somalia OR South Africa OR St Helena OR Sudan OR Swaziland OR Tanzania OR Togo OR Tunisia OR Uganda OR “Western Sahara” OR Zaire OR Zambia OR Zimbabwe OR “Central Africa” OR “Central African” OR “West Africa” OR “West African” OR “Western Africa” OR “Western African” OR “East Africa” OR “East African” OR “Eastern Africa” OR “Eastern African” OR “North Africa” OR “North African” OR “Northern Africa” OR “Northern African” OR “South African” OR “Southern Africa” OR “Southern African” OR “sub Saharan Africa” OR “sub Saharan African” OR “subSaharan Africa” OR “subSaharan African”) NOT (“guinea pig” OR “guinea pigs” OR “aspergillus niger”)). The search phrase for the African countries was adapted from a search phrase developed by researchers Pienaar et al. (2011) [65].

Inclusion/exclusion criteria:

- Exclusion criteria
  - Studies concerning a different parasite than *T. gondii*;
  - Studies on *T. gondii* in domestic animal species;
  - Studies reporting/using data older than 1900 or published after 31 December 2020;
  - Studies reporting results from outside the study area;
  - Studies reporting results out of the scope of the review question.
  - Duplicate records.
- Inclusion criteria
  - Studies reporting data on T. gondii from the African continent in both free ranging or captive wild species.

Variables extracted: Authors, year of publication, country, animal species, number of animals sampled, number of animals positive and detection method. Data were entered in Excel sheets.

Languages: English articles.

Study period: 1 January 1990–31 December 2020

Geographical range: All countries within the African continent.

## Appendix B

**Table A1:** PRISMA checklist.

Section and Topic	Item #	Checklist Item	Location Where Item is Reported
TITLE			
Title	1.	Identify the report as a systematic review.	Page 1
ABSTRACT			
Abstract	2.	See the PRISMA 2020 for Abstracts checklist.	Page 1
INTRODUCTION			
Rationale	3.	Describe the rationale for the review in the context of existing knowledge.	Pages 1–2
Objectives	4.	Provide an explicit statement of the objective(s) or question(s) the review addresses.	Pages 1–2
METHODS			
Eligibility criteria	5.	Specify the inclusion and exclusion criteria for the review and how studies were grouped for the syntheses.	Pages 12–13
Information sources	6.	Specify all databases, registers, websites, organisations, reference lists and other sources searched or consulted to identify studies. Specify the date when each source was last searched or consulted.	Pages 12–13
Search strategy	7.	Present the full search strategies for all databases, registers and websites, including any filters and limits used.	Pages 12–13
Selection process	8.	Specify the methods used to decide whether a study met the inclusion criteria of the review, including how many reviewers screened each record and each report retrieved, whether they worked independently, and if applicable, details of automation tools used in the process.	Pages 12–13
Data collection process	9.	Specify the methods used to collect data from reports, including how many reviewers collected data from each report, whether they worked independently, any processes for obtaining or confirming data from study investigators, and if applicable, details of automation tools used in the process.	Pages 12–13
Data items	10. (a)	List and define all outcomes for which data were sought. Specify whether all results that were compatible with each outcome domain in each study were sought (e.g., for all measures, time points, analyses), and if not, the methods used to decide which results to collect.	Pages 12–13
	10. (b)	List and define all other variables for which data were sought (e.g., participant and intervention characteristics, funding sources). Describe any assumptions made about any missing or unclear information.	Pages 12–13
Study risk of bias assessment	11.	Specify the methods used to assess risk of bias in the included studies, including details of the tool(s) used, how many reviewers assessed each study and whether they worked independently, and if applicable, details of automation tools used in the process.	Pages 11–13
Effect measures	12.	Specify for each outcome the effect measure(s) (e.g., risk ratio, mean difference) used in the synthesis or presentation of results.	Pages 11–13
Synthesis methods	13. (a)	Describe the processes used to decide which studies were eligible for each synthesis (e.g., tabulating the study intervention characteristics and comparing against the planned groups for each synthesis (item #5)).	Pages 12–13
	13. (b)	Describe any methods required to prepare the data for presentation or synthesis, such as handling of missing summary statistics, or data conversions.	Pages 12–13
	13. (c)	Describe any methods used to tabulate or visually display results of individual studies and syntheses.	Pages 12–13
	13. (d)	Describe any methods used to synthesize results and provide a rationale for the choice(s). If meta-analysis was performed, describe the model(s), method(s) to identify the presence and extent of statistical heterogeneity, and software package(s) used.	Not applicable
	13. (e)	Describe any methods used to explore possible causes of heterogeneity among study results (e.g., subgroup analysis, meta-regression).	Not applicable
	13. (f)	Describe any sensitivity analyses conducted to assess robustness of the synthesized results.	Not applicable
Reporting bias assessment	14.	Describe any methods used to assess risk of bias due to missing results in a synthesis (arising from reporting biases).	Pages 11–13
Certainty assessment	15.	Describe any methods used to assess certainty (or confidence) in the body of evidence for an outcome.	Pages 11–13
RESULTS			
Study selection	16. (a)	Describe the results of the search and selection process, from the number of records identified in the search to the number of studies included in the review, ideally using a flow diagram.	Pages 2–11
	16. (b)	Cite studies that might appear to meet the inclusion criteria, but which were excluded, and explain why they were excluded.	Pages 11–12
Study characteristics	17.	Cite each included study and present its characteristics.	Pages 2–11
Risk of bias in studies	18.	Present assessments of risk of bias for each included study.	Pages 2–11
Results of individual studies	19.	For all outcomes, present, for each study: (a) summary statistics for each group (where appropriate) and (b) an effect estimate and its precision (e.g., confidence/credible interval), ideally using structured tables or plots.	Page 11
Results of syntheses	20. (a)	For each synthesis, briefly summarise the characteristics and risk of bias among contributing studies.	Pages 2–11
	20. (b)	Present results of all statistical syntheses conducted. If meta-analysis was done, present for each the summary estimate and its precision (e.g., confidence/credible interval) and measures of statistical heterogeneity. If comparing groups, describe the direction of the effect.	Pages 2–11
	20. (c)	Present results of all investigations of possible causes of heterogeneity among study results.	Pages 2–11
	20. (d)	Present results of all sensitivity analyses conducted to assess the robustness of the synthesized results.	Pages 2–11
Reporting biases	21.	Present assessments of risk of bias due to missing results (arising from reporting biases) for each synthesis assessed.	Page 11
Certainty of evidence	22.	Present assessments of certainty (or confidence) in the body of evidence for each outcome assessed.	Pages 2–11
DISCUSSION			
Discussion	23. (a)	Provide a general interpretation of the results in the context of other evidence.	Pages 9–11
	23. (b)	Discuss any limitations of the evidence included in the review.	Page 11
	23. (c)	Discuss any limitations of the review processes used.	Page 11
	23. (d)	Discuss implications of the results for practice, policy, and future research.	Pages 9–13
OTHER INFORMATION			
Registration and protocol	24. (a)	Provide registration information for the review, including register name and registration number, or state that the review was not registered.	Not applicable
	24. (b)	Indicate where the review protocol can be accessed, or state that a protocol was not prepared.	Page 12
	24. (c)	Describe and explain any amendments to information provided at registration or in the protocol.	Pages 14–16
Support	25.	Describe sources of financial or non-financial support for the review, and the role of the funders or sponsors in the review.	Page 13
Competing interests	26.	Declare any competing interests of review authors.	Page 13
Availability of data, code and other materials	27.	Report which of the following are publicly available and where they can be found: template data collection forms; data extracted from included studies; data used for all analyses; analytic code; any other materials used in the review.	Page 13
